# Investigating the impact of emotion on temporal orientation in a deep multitask setting

**DOI:** 10.1038/s41598-021-04331-3

**Published:** 2022-01-11

**Authors:** Sabyasachi Kamila, Mohammad Hasanuzzaman, Asif Ekbal, Pushpak Bhattacharyya

**Affiliations:** 1 Department of Computer Science and Engineering, Indian Institute of Technology Patna, Patna, India; 2 Department of Computer Science, Munster Technological University (Cork Campus), Cork, Ireland; 3 Department of Computer Science and Engineering, Indian Institute of Technology Bombay, Mumbai, Maharashtra India

**Keywords:** Psychology, Human behaviour

## Abstract

Temporal orientation is an important aspect of human cognition which shows how an individual emphasizes past, present, and future. Theoretical research in psychology shows that one’s emotional state can influence his/her temporal orientation. We hypothesize that measuring human temporal orientation can benefit from concurrent learning of emotion. To test this hypothesis, we propose a deep learning-based multi-task framework where we concurrently learn a unified model for temporal orientation (our primary task) and emotion analysis (secondary task) using tweets. Our multi-task framework takes users’ tweets as input and produces three temporal orientation labels (*past*, *present* or *future*) and four emotion labels (*joy*, *sadness*, *anger*, or *fear*) with intensity values as outputs. The classified tweets are then grouped for each user to obtain the user-level temporal orientation and emotion. Finally, we investigate the associations between the users’ temporal orientation and their emotional state. Our analysis reveals that *joy* and anger are correlated to *future* orientation while *sadness* and *fear* are correlated to the *past* orientation.

## Introduction

The emergence of digital data revolutionized research in the area of social science. Several human attributes including age, gender, education, psychological well-being, etc. can be predicted and analyzed using different social media data like tweets, Facebook posts, etc^1–5^. In this context, human temporal orientation is an emerging area of research at the cross-section of Natural Language Processing, Machine Learning, and Social Science where social media texts can be utilized efficiently to measure one’s temporal orientation.

Human Temporal orientation refers to a cognitive operation which shows how an individual emphasizes *past*, *present* and *future*^6^. How individuals differ in their temporal orientation and what reasons lead them to do so can show their future goal-setting, health, and education^6–8^. Studies in psychology and social science reveal that temporal orientation has a huge impact on our behavior, interpersonal relation, emotion, health, attitudes, educational achievements, sexual behavior, sleep and dreaming patterns, academic goal setting, risk-setting, etc^6,7,9–13^. Previous psychological studies also revealed that human temporal orientation can be associated with other attributes such as age, education, gender, happiness, anger, depression, anxiety and aggression^14–18^.

Traditionally human temporal orientation is measured by self-report questionnaires. However, language-based assessments can be used to study human temporal orientation as an alternative to the questionnaire-based approach^19–21^. Twitter data has been a prior choice for language-based study as it is less costly and easily accessible for research purposes^22,23^. Tweets are noisy containing many ungrammatical constructions which makes it a very challenging text form to handle^24,25^. Thus our choice of considering Twitter data for this study is backed up by easy accessibility, challenges, and potential information for language-based studies.

Recent studies show that temporal orientation can be measured based on human-written texts^20,21,26,27^. For example, *‘I hope for a better world.’* has a *future* temporal orientation while the text, *‘My childhood days are the best days of my life.’* has a *past* temporal orientation. All these methodologies focus on the performance improvement of single-task learning models, by better characterizing temporal orientation itself. However, some psychological research works show that human temporal orientation can be associated with one’s emotional states^28,29^. For example, *joy* has been related to *future* orientation^30^ while *sadness* has been related to *past* and *present* orientation^31,32^. For illustration, the sentence *‘I am very excited about the upcoming movie release’* has a temporal orientation as *future* and emotion as *joy*. Another sentence, *‘I did everything but I failed’* has a temporal orientation as *past* and emotion as *sadness*. As a consequence, we hypothesize that the measurement of human temporal orientation can benefit from the concurrent learning of human emotion.

The focus of this current study is two-fold. The first fold examines whether the performance of the tweet-level temporal orientation improves by using emotional signals in the tweets. We formulate the problem in a multi-task learning framework that simultaneously learns the temporal orientation and emotion of tweets. Here, temporal orientation classification is our primary task, and emotion analysis is our auxiliary task. We jointly learn a unified model from the shared representations of all the tasks, expecting that each will benefit from the other, and perform better compared to a single-task setting, where the tasks are performed in isolation. The second fold aims for quantifying the person-level temporal orientation in a large-scale empirical manner and find a relation with the person-level emotion. Existing person-level association between human temporal orientation and emotion mostly is in the space of psychology in limited settings (limited number of participants with a particular age distribution, for example, graduate students of an institute).

Our tweet-level method follows a two-layer framework where in the first layer we build a weakly labeled training set containing three temporal (*past*, *present* or *future*) categories via a *Generative Task* without using any hand-labeled annotations. There is no existing large gold standard training set (manually annotated) for temporal orientation tasks. In this regard, researchers built weakly-labeled training sets using limited heuristic rules (keyword-based^20^, hashtag-based^21^). These approaches do not consider any statistical approach for labeling. Our approach, in contrast, uses both heuristics (human knowledge) as well as statistical approaches (ML models) and uses an optimization technique for generating final labels.

The second layer of the framework is a *Discriminative Task* where, in a multi-task setting, we simultaneously predict three task outputs (temporal orientation, emotion class, and emotion intensity). For emotion classification, we use two set of data from the well-known SemEval Task 1^33^ where the training set and test set are manually annotated with four emotional classes, such as *joy*, *sadness*, *anger* and *fear*. As the emotion data has only four classes we could only consider those categories for our experiment.

At user-level (person-level), we use our multi-task model to predict 5,191 Twitter users’ (UK population) $\approx$10 million tweets developed by^34^. We then group the tweet-level temporal orientation and emotion measure over users to obtain user-level measures. Finally, we investigate the relationship between user-level temporal orientation and emotion.

The main contributions of this article are summarized as follows:

- we put the hypothesis on a test that temporal orientation can benefit from the concurrent learning of human emotion.
- we create a temporal orientation training set without using any hand-labeled annotations.
- we propose a deep multi-task model to jointly learn temporal orientation and emotion (class and intensity) from the tweets. The model attains improvements over the baselines.
- we investigate the relationship between the user-level temporal orientation and emotion in a large-scale empirical manner.

## Related background

Research on Temporal Orientation evolved in the field of psychological time which expresses personal involvements and concentrations on the *past*, *present* and *future*^35–37^. The structural (cohesion, span, and direction) aspects of temporal orientation focused on defining temporal orientation and investigating different socio-cultural aspects associated with it^38,39^ while the functional aspects of it concentrated on programming one’s actions in time which include the effectiveness of time usage as well as its consequences. Such theoretical methods aim to measure temporal orientation by the means of questionnaires. Theoretical temporal orientation measure has been done by many researchers but more predominantly by Zimbardo Time Perspective Inventory (ZTPI)^7^ and Consideration of Future Consequences scale (CFC)^40^.

Past researches have shown that temporal orientation has an impact on education, health, psychological well-being, risk-taking, organizational behavior, etc.^9–13^. Holman et al.^41^ have shown that *past* temporal orientation is related to long-term distress in individual who experienced trauma. Research shows that high *future* orientation leads to safer sex^42^, helps in academic goal-setting^10^ and better strategic plannings^43^. Future oriented people use less tobacco, have lower body mass index, do more physical exercises, and save money for future^16^. Brown et al.^44^ have shown that *present* oriented people felt significantly less susceptible to consequences of uncontrolled hypertension. Present oriented people were also found to have more suicidal tendency than the *future* oriented people^45^.

Recent large-scale empirical studies using social media data have measured human temporal orientation from texts^19–21,27,46^. In these researches, machine learning-based classification models are built for predicting temporal orientation. The authors then investigated the association between user-level temporal orientation and various user-level attributes such as age, gender, Big-five personality, IQ, satisfaction with life, income level, relationship status, education, intelligence, and optimism. All of these works were performed in a single task setting while our current study proposes a multi-task learning framework where a single model is jointly learned for temporal orientation and emotion tasks.

Many earlier works considered focus on emotion detection from texts using different machine learning techniques^47,48^. Dedicated tasks like SemEval^49^, WASSA-2017^50^ incorporated emotion detection tasks with four emotion categories, namely *joy*, *sadness*, *anger* and *fear*. Different emotion related lexicons, such as NRC-Emotion lexicon^51^, EmoBank^52^ etc., were created to further facilitate research in this direction. Few authors used multi-task learning considering different emotion categories as different tasks^53^. Our current research is completely different from these existing works in the sense that we, for the very first time, attempt to develop a deep multi-task framework for detecting temporal orientation with the help of emotional information.

Besides, there have been prior works that intended for solving particular tasks like political argument extraction, handling misinformation, rumour detection, etc using tweets. The authors in^54^, presented a study that shows that there is a political argument on Twitter and the quality of public argument can be communicated by speech acts in tweets. In^55^, the authors presented a trend analysis of misinformation spread on Twitter due to COVID-19 and resolved few implications of it. In^56^, the authors proposed a Generative Adversarial Network-based approach to detect rumours with explanations from tweets. The study reported in^57^ investigated whether the social media reaction to the COVID-19 pandemic in three critically affected countries has significant relations with their observed mortality a month later. In contrast to all these studies, we make use of multi-task learning to simultaneously learn temporal orientation and emotion from tweets. The user-level data^34^ that we use are of 5191 Twitter users of the UK population. In the data, the tweeters were analysed before collecting data. The authors adopted a standardised job classification taxonomy (a UK government system developed by the Office of National Statistics for classifying occupations)^58^ for mapping Twitter users to occupations. The Twitter users were mapped to their occupation along with their historical tweets and profile information. The users are well balances between different occupational groups. The tweets were not used for any specific social phenomena. Rather the users were selected randomly based on the mapping of occupational classes.

## Results

We divide this section into three subparts. In the first part, we analyze the performance of the generative task. The second part shows the result of the multi-task discriminator framework and the final part investigates the relationship between user-level temporal orientation and their emotion by measuring the correlation between them.

### Results of generation task

To validate the quality of the generated training set for temporal orientation, we manually annotated 500 samples, randomly collected from the training set, and checked against the automatically generated labels using our generative task. We find our label generative model as of acceptable quality (75.23% accuracy). The results in Table 1 show the individual performance of each weak label-generators. We achieve accuracies of 66.55%, 65.23% and 69.54% when we use only Heuristic (a), (b) and (c) (cf. Section **Heuristic rules**), respectively for label generation. We achieve the accuracy of 72.30% and 74.11% when we use only weak model SVM, and weak model B-LSTM (cf. Section **Weak Models**), respectively. Our proposed generative model achieves the highest performance (75.23% accuracy) when we use all the three rules along with the two weak models to generate final probabilistic labels.

**Table 1:** Accuracy (in %) on validation data for label generation task for creating optimised temporal orientation labels in training set.

Heuristic(a)	Heuristic(b)	Heuristic(c)	Weak_SVM	Weak_B-LSTM	Proposed generative model
66.55	65.23	69.54	72.30	74.23	**75.23**

Heuristic(a): keyword-based weak-label generation, Heuristic(b): Knowledge-based weak-label generation, Heuristic(c): verb-based weak-label generator, Weak_SVM: weak-labels generated by SVM classifier, Weak_BLSTM: weak-labels generated by BLSTM classifier.

### Results of multi-task classification

**Figure 1:**
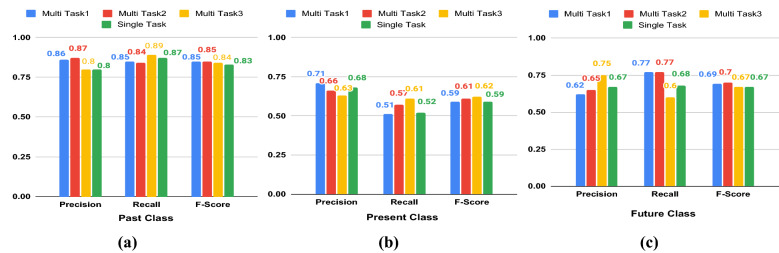
Comparative results of single task and multitask temporal orientation classification. Class-wise Precision, Recall, and F1-measure shown for **(a)** Present class, **(b)** Present class, **(c)** Future class. Multi Task1: when emotion dataset used is SemEval-18 Task 1,EI-OC, Multi Task2: when emotion dataset used is SemEval-18 Task 1, EI-REG. Multi Task3: when emotion dataset used is Tweet Covid-19.

**Figure 2:**
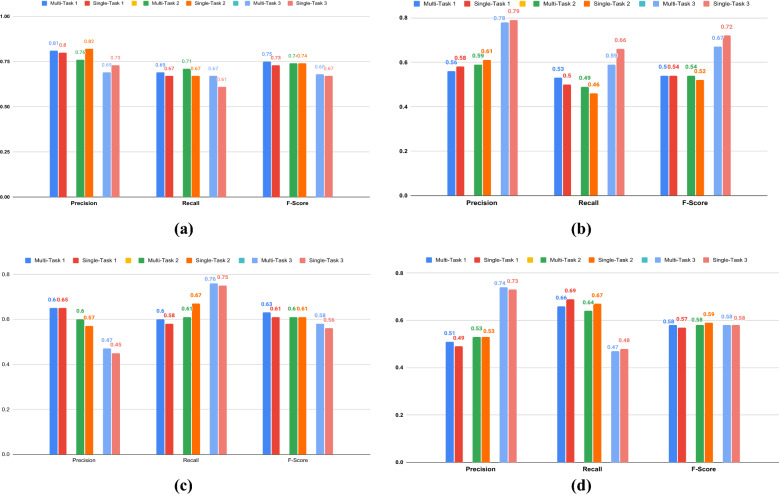
Comparative results of single task and multitask emotion classification. Class-wise Precision, Recall, and F1-measure shown for **(a)** Joy class, **(b)** Sadness class, **(c)** Anger class, **(d)** Fear class. Multi Task1, Single Task 1: when emotion dataset used is SemEval-18 Task 1,EI-OC, Multi Task2, Single Task 2: when emotion dataset used is SemEval-18 Task 1, EI-REG. Multi Task3, Single Task: when emotion dataset used is Tweet Covid-19.

We report the results of the multi-task classification in Table 2. The performance of the emotional intensity is measured by the Pearson correlation coefficient *r* between the original score and the predicted score. For temporal orientation classification, our multi-task models achieve highest accuracies of 75.30%, 75.98% and 75.17% (Table 2). For the emotion analysis, the multi-task models achieve 62.29% accuracy and r $=$ 0.49 (using EI-oc data) and 63.05% accuracy and r $=$ 0.55 (using EI-reg data) and 62.81% accuracy and r $=$ 0.57 (using tweet covid-19 data). We see a performance improvement of all the tasks in the multi-task setting from the single-task setting. The class-wise performances (precision, recall, F1-score) of temporal orientation and emotion analysis tasks are reported in Figs. 1, and 2, respectively. We also compare our models (single-task and multi-task) with the state-of-the-art (SOTA) single task temporal orientation model (accuracy of 72.20%). For temporal orientation, we consider the baseline model as the method proposed in^21^. The baseline for original SemEval Task 1 (EI-oc, and EI-reg)^33^ were different as the tasks were to predict the emotional intensity of a tweet given the class of the tweet. Thus, we can not directly compare their results.

For this reason, here we report SVM-based baseline results for the single-task emotion classification. As there is no existing work that considers temporal orientation in a multi-task setting, we consider a recent framework^59^ and use our dataset to make a multi-task baseline. The framework integrated a pooling layer into a Bi-directional Recurrent Neural Network for each task. Then the pooling outputs of the tasks are shared and an attention mechanism between the shared layers and the task-specific layers is used to get the shared features. The results are reported in Table 2.

We observe that both, our single task and multi-task frameworks for temporal classification, beat the SOTA performance (both single-task and multi-task). For the emotion analysis, our multi-task method performs a little better (accuracy of 63.05%, r=0.55) compared to the single-task method (accuracy of 62.07%, r=0.42) as well as the baseline (accuracy of 60.08%, r=0.32 for the single-task baseline and accuracy of 61.55%, r=043 for the multi-task baseline) for the emotion analysis using EI-OC data (Table 2). We see the same pattern when we use the other emotion data, i.e. EI-reg and Tweet covid-19 (Table 2). We believe that our multi-task method is of acceptable quality as our primary task is the temporal orientation while we portray the emotion analysis as an auxiliary task. We perform a Statistical t-test^60^ and find that the performance improvement in our proposed multi-task models over the single-task models as well as the baselines is statistically significant (p < 0.001).

**Table 2:** Comparative performance (% accuracy for classification and Pearson correlation coefficient for intensity) on the manually annotated test set for multi-task and single task learning.

		Method	Multi-task			Single-task		
			Temporal orientation	Emotion analysis		Temporal orientation	Emotion analysis	
				Class	Intensity		Class	Intensity
**Dataset**	SemEval-18 Task 1, EI-OC^33^	Baseline	73.05	61.55	0.43	72.20	60.08	0.32
		Proposed	75.30	62.29	0.49	74.08	61.35	0.36
	SemEval-18 Task 1, EI-REG^33^	Baseline	73.67	62.61	0.44	72.20	61.23	0.40
		Proposed	**75.98**	**63.05**	0.55	74.08	62.07	0.42
	Tweet Covid-19^61^	Baseline	72.98	62.12	0.52	72.20	61.89	0.53
		Proposed	75.17	62.81	**0.57**	74.08	62.63	0.55

Temporal orientation single-task baseline: work proposed in^21^. Multi-task baseline: architecture proposed in^59^.

#### Result analysis

We manually analyze the performance of our method and found strong signals in support of our hypothesis. Precisely, we checked the instances where multi-task and single-task models differed. We noticed that the multi-task model tends to perform better for tweets having implicit temporal signals. For example, the tweet *‘I miss those days so badly.’* has underlying temporal orientation as *past*. Our single task model predicts it as *future* while the multi-task model correctly predicts it as *past*. The emotion involved in the tweet is ’*sadness*’ which has been related to *past* orientation^31,32^ in psychological literature. Here, emotion adds an extra signal to the model which helps to decide temporal orientation. Another example, ‘Lightning in the eastern sky look like they may add to firework-y excitement’ has a temporal orientation as *future*, our single task model fails and classifies it as *past* but our multi-task does not and correctly classify it as *future*. The reason for this is the presence of a joyful word ‘excitement’ which adds a signal to the model (*joy* has been related to *future* orientation in the literature^30^). Confusion matrix shows that our multi-task model mostly misclassifies *present* tweets into *future*. For example, the tweet ‘*Good evening Here we go.*’ is *present* oriented but our multi-task model predicts it as *future* due to a joyful signal associated with it.

## Discussion

**Figure 3:**
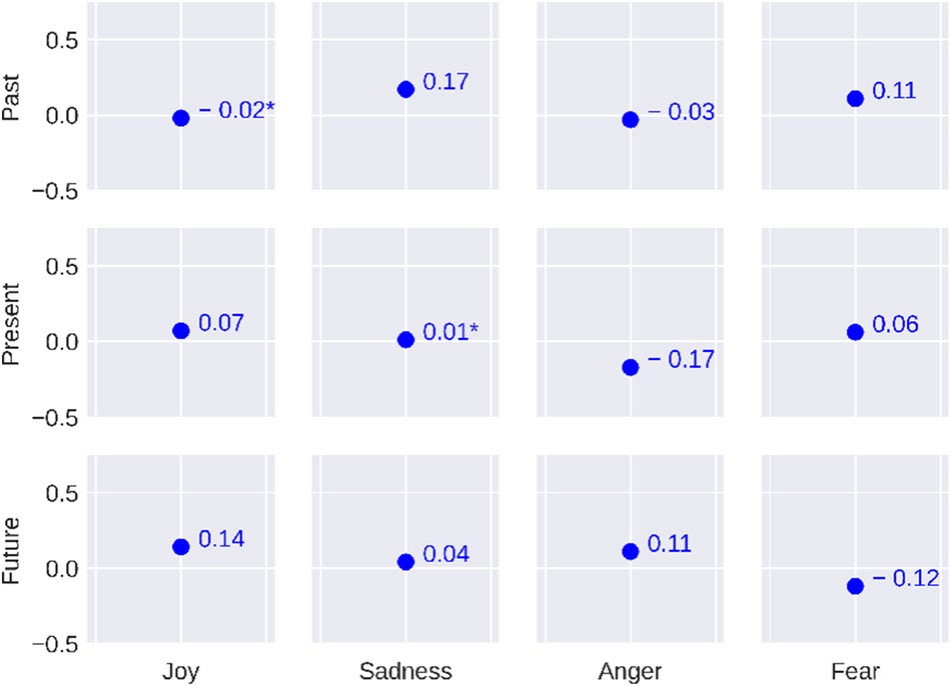
Correlation between the users’ temporal orientation and emotion. (*) before any value signifies that those values are not statistically significant.

**Figure 4:**
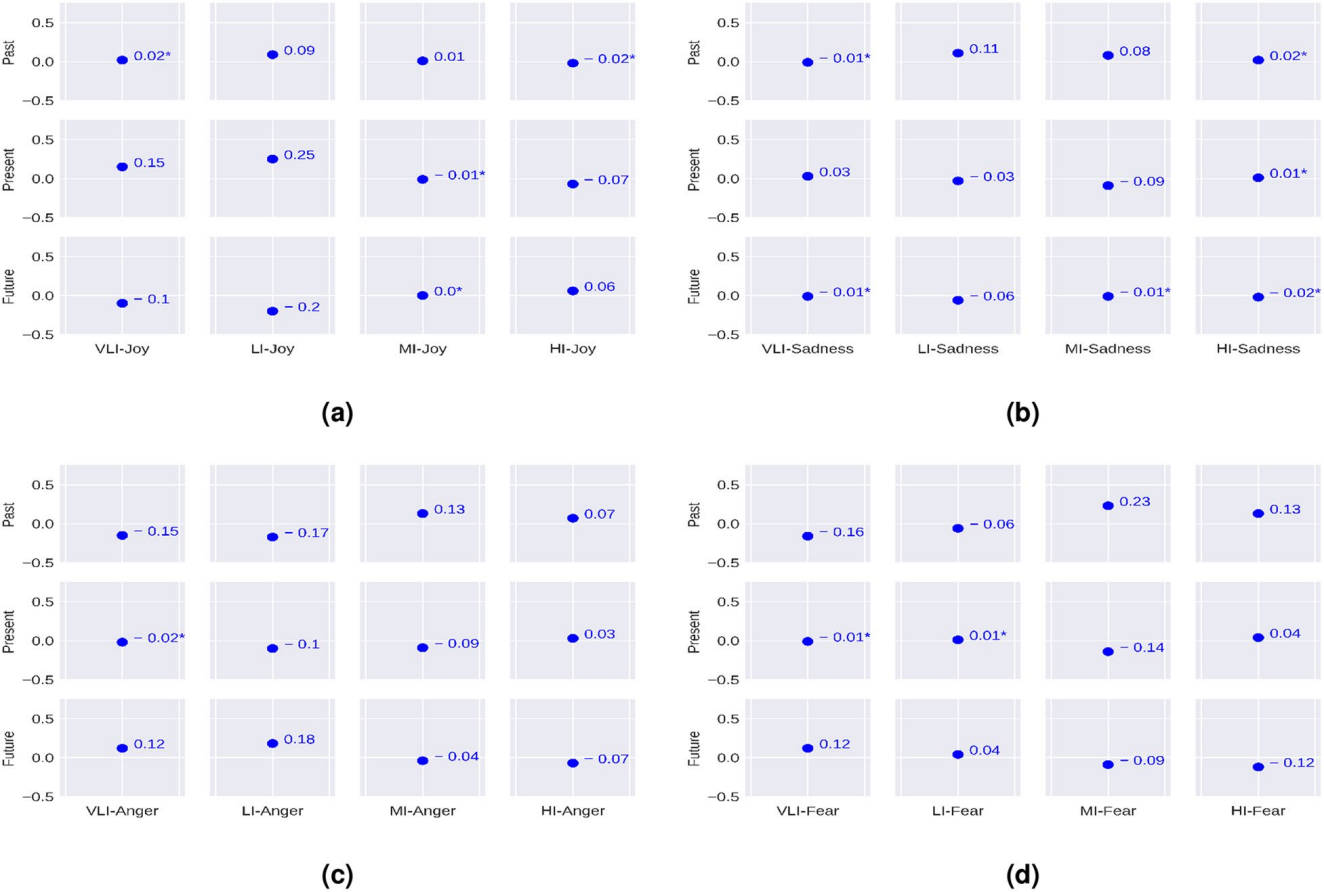
Correlation between the users’ temporal orientation and emotion intensity. **(a)** Correlation between temporal orientation and different intensities of Joy, **(b)** correlation between temporal orientation and different intensities of Sadness, **(c)** correlation between temporal orientation and different intensities of Anger, **(d)** correlation between temporal orientation and different intensities of Fear. Here, *VLI* very low intensity, *LI* low intensity, *MI* moderate intensity, *HI* high intensity. (*) before any value signifies that those values are not statistically significant.

Here, we investigate the associations between the user-level temporal orientation and emotion in terms of the Pearson correlation coefficient *r*. The correlations were measured by the methods mentioned in Section . We perform McNemar’s test^62^ and the results reported below are statistically significant (p<0.05). The results are reported in Figs. 3 and 4.

We observe in Fig. 3 that *future* temporal orientation has a positive correlation with *joy* (*r*$=$ 0.14) which suggests that future-focused people are more joyful which is in line with the psychological literature^30^. In psychological literature *sadness* has been related to *past* and *present* orientation^31,32^. Our empirical analysis shows that *sadness* is positively correlated to *past* orientation (*r* $=$ 0.17).

In psychological literature, *anger* has been related to the time-bound factor which in-tern related to the *future* orientation^8^. Another research suggests that *present* focus may be associated with *anger*^63^. Our results reveal that *anger* has a positive correlation with the *future* orientation (*r* $=$ 0.11) and a negative correlation with the *present* orientation (*r* $= -0.17$). Literature suggests that fear arises based on any bad past events which an individual had to face^64^. We find that *past* orientation has a positive correlation with *fear* (*r* $=$ 0.11) while *future* orientation has a negative correlation with *fear* (*r*=− 0.12).

In Fig. 4, we report correlation between temporal orientation and different emotional intensity values. We observe that the past orientation is related to low-intensity joy, low-intensity sadness, moderate-intensity anger, and high-intensity fear. The present orientation has a positive correlation with low-intensity joy, and a negative correlation with low-intensity anger and moderate-intensity fear. We also find that the future orientation has a positive correlation with high-intensity joy, low-intensity anger, and very low-intensity fear.

Our measurement of the temporal orientation is for a large number of users. In literature, where temporal orientation was measured on a large scale using the social media data, we see that low correlation coefficient values have made a significant contribution^19,21^. The statistical method needs context. Since we do not have and do not expect to have breakthroughs via Twitter data-driven approach, low correlation coefficient values carry significance in the context of this current study.

Although we have backed our empirical findings with psychological references, here we report some limitations which may require more future research in this direction. Firstly, we use a particular data set of Twitter which we believed to have potential information for studying the language-based analysis (here temporal orientation). It would have been better if we could also calculate the self-report measure of the temporal orientation of these Twitter users and compare them with the language-based assessments. However, the existing user-level data is anonymous (for ethical concern) and the number of users is large (5191). So, the self-report study is not feasible for a large number of users. Whether using another type of data (Blogs, news articles) can vary the measurement and relationship is a matter of further research.

Secondly, the user-level data is of the UK population and socio-cultural differences have effects on the temporal orientation. In psychological literature, we see that people’s age, gender, education and other factors influence the temporal orientation^9–13^. For example, females are more future-oriented than males. So, if the dataset has more samples of females than males then it will be biased towards the future orientation. Similarly, if people of only a young age group are considered then it will also be biased towards the future as young people are more future-oriented. The temporal orientation also varies over culture. For example, people in some cultures are more future-oriented and some are more past-oriented. It is also true for emotion. Our user-level Twitter dataset has ages distributed between 10 and 60, where males and females are almost balanced. However, the data is of the UK population and the measurements may vary if we could use a dataset of the other socially or culturally different regions.

Finally, we also believe that more fine-grained aspects of human emotion can add better signals to measure the temporal orientation. Although more fine-grained emotion detection from texts would be a very difficult task and can add more errors when mapped to large-scale data. It is possible to have more than one temporal or emotion tag in a tweet. Those tweets will get a single tag based on the current model and dataset. As we don’t have a dataset for the multi-label temporal orientation we place this in the scope of future work. We also agree that the context of the tweet matters, but the contextual information is not available in any of the datasets. So, we have to rely on the tweet itself, i.e. hashtag information, mentions, keywords, or the implicit meaning of the tweets to resolve contexts. However, the data is well-chosen and annotated with experts. Thus we believe that it has much information for the machine learning model to learn.

From our experiments, we observe that emotional information helps the temporal orientation in the multi-task setting. Multi-task learning is useful when one task has more data available and another task has limited data and there is a dependency between the two tasks. In the current scenario, there are lots of data (marketing strategy, review data, etc) available for the emotion analysis but limited in the case of temporal orientation. Both temporal orientation and emotional impact on applications like compulsive vs impulsive buying, political, financial aspects, author profiling, etc. Natural language applications in commerce, public health, disaster management, and public policy can benefit from knowing the affectual states of people-both temporal orientation, and the categories and intensities of the emotions they feel.

The valence (positive vs negative) and arousal (high arousal/alertness vs low arousal/drowsiness) of tweets have the ability to widen the scope of our present work. Here are a few examples:- ‘bored’ is a low arousal state that is usually fairly negative (or neutral); ‘anger’ and ‘fear’ express a high arousal negative state; while ‘sadness’ is a low arousal negative state, ‘excitement’ is a high arousal positive state; ‘content’ is a low or neutral arousal and positive state and so forth. The temporal orientation may have more nuances which can be grasped by glancing at valence and/or arousal first and then more fine-grained emotion classes. As our present model is not discussing this because of the unavailability of such tags in the dataset, new findings can be obtained when emotions are models in different ways. To exemplify this, in our current work a tweet about ‘failure’ is taken in the class ‘sadness’, but it can also elicit some more emotions, comprising social emotions (e.g. shame, embarrassment), which are not encompassed by our current model. This may guide us to sum up that ‘sadness’ may have other types of emotional differences which are the real basis for its correlation with the past orientation. We look for the proper dealing of these with a better data-set with more fine-grained emotion groups.

## Methods

We divide our method into two parts: the first part describes tweet-level temporal orientation and emotion analysis and the second part describes the user-level analysis of temporal orientation and emotion.

### Tweet-label measurement of temporal orientation and emotion

Here, we propose a generative-discriminative framework where the generator generates optimized labels of training set for temporal orientation classification. Our discriminator is a multi-task deep learning framework where we simultaneously predict the temporal orientation and emotion of a tweet. Here, we use an existing dataset for emotion classification. We then use this model to label the tweets of 5,191 Twitter users into either of *past*, *present* or *future* classes and either of *joy*, *sadness*, *anger* or *fear* classes. Finally, we aggregate the tweet label temporal orientation and emotion measures to get user-level assessments. Thereafter, we measure the correlation between user-level temporal orientation and emotion.

#### Generative task

**Figure 5:**
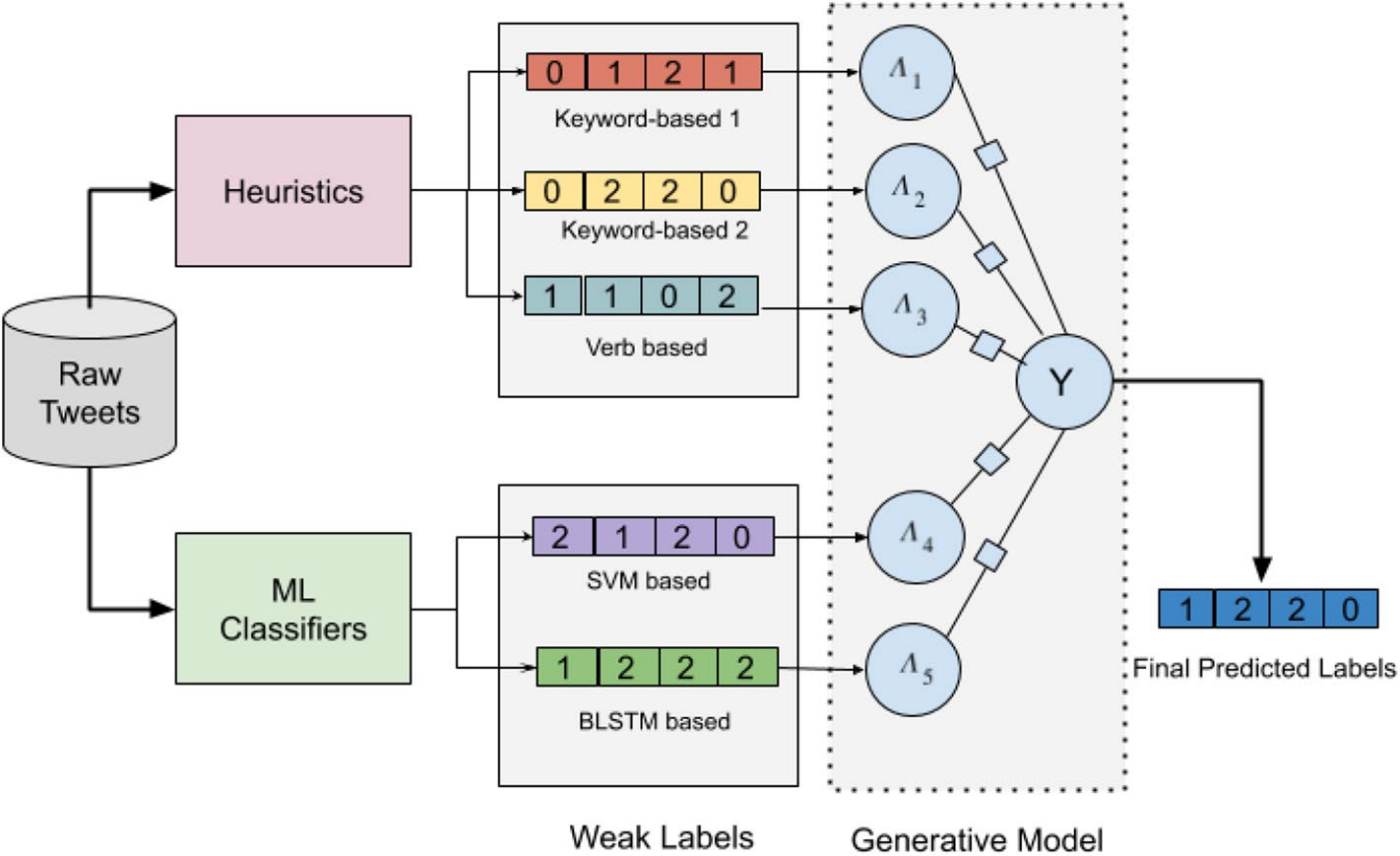
Proposed label generative task.

In any classification task, quality training data labels are crucial for creating a better model. In this study, for generating training data labels we investigated different weak supervision techniques. Weakly supervised techniques analyze different heuristic rules, external knowledge-base, crowd-sourced annotations, or existing statistical models to generate labels of samples computationally. In this case, the need for manually labeling the samples becomes obsolete. Thus data generated this way is very cheaper. However, this approach suffers from low coverage due to a limited number of rules thus resulting in low accuracy. To improve the accuracy and enhance the coverage, the generative model combines the weak labels by optimization technique to generate final optimized labels. We depict the architecture in Fig. 5.

#### Label generation

In this study, from raw tweets, we assign different weakly labels of either of *past*, *present* or *future* temporal orientation classes using different heuristics and weak models. In heuristic-based techniques, weakly supervised labels are generated computationally by analyzing different rules whereas statistical weak models exploit existing temporal orientation models to create weak labels. We describe the details of these two weak label generation techniques below.

#### Heuristic rules

The underlying idea of defining heuristic rules is that if a tweet contains at least one word of a particular temporal class then we assign a weak label of that temporal category to the tweet. Depending upon how we obtain the temporal orientation of a word can assign different weak labels to a single tweet. We choose three such ways as (a) obtain the temporal orientation of the words from a temporal keywords list created by Schwartz et al.^26^, (b) from a bunch of *past*, *present* and *future* oriented keywords (30 from each category) created by Hasanuzzaman et al.^20^ and (c) from the Part-of-Speech (PoS) tags of each word of a tweet where the tags are created using the CMU tweet tagger^65^. These three weakly supervised label sets are then encoded to the generative model as depicted in Fig. 5.

####  Weak models

The underlying idea of weak models is that we use existing trained temporal orientation models to generate weak labels of tweets. We use two models like Support Vector Machine (SVM)^66^, and Bidirectional Long Short Term Memory (B-LSTM)^67^ which is trained using an existing temporal labeled training set^21^ and the trained models are then used to generate weak labels of the raw tweets.

#### Label optimization

The core concept of the generative model is label optimization. After generating different weak labels following heuristic rules and weak models, these labels are fed to the developed generative model. The generative model finally assigns a single label (*past* or *present* or *future*) for each tweet. The major issue here is how to combine all these weak labels because the correlations between those weak labels as well as the correctness and quality of each weak model are not known. This phase is censorious and the generative model plays an important role to resolve this.

We develop a generative model that optimizes the weak labels to produce a single label for each tweet. This generative model estimates a structure to accurately resolve the correlation between the weak labels. To accomplish this, we use a structure estimation method^68^. The concept of structure learning is well studied in the supervised setting. The structure learning for weak supervision is challenging because the true class labels are latent. Also, the supervised sources are not conditionally independent which means there are statistical dependencies among the sources. Thus resolving these dependencies to give a single label to a text becomes important. The same challenges exist for our task as in temporal orientation prediction from texts, weak labeling rules do overlap.

By considering these dependencies, the generative model enhances its predictive capabilities. For example, if some of the labeling functions use similar kinds of pattern matching rules then this dependency can be included in the model. These pair-wise correlations are important and should be considered^69^. The structure estimation method selects a set *S* containing all labeling function pairs (q, r) as a means of correlations. Then the generative model can be represented as a factor graph $\mathbb {G}$, a probabilistic graphic model. This model consists of two types of nodes, evidence variable, and factors. The factors node in $\mathbb {G}$ represents the relationship between variables to be estimated. All the weak labels are represented as evidence variables of $\mathbb {G}$. In our case, these weak labels are the three labels say, L1, L2, and L3 acquired using three heuristics as well as two weak model-based labels $L_{SVM}$, and $L_{BLSTM}$.

If there are a total of *L* such labeling functions and a total of *M* number of tweets, then we create a label matrix $\Lambda \in (0, 1, 2, ...S)^{M \times L}$ which the generative model takes as input. Our final generative model is represented as $\rho _{z}(\Lambda , Y)$ (Here, Y is a set of all possible labels and z is the parameter to be estimated). The generative model uses three-factor dependency types namely, accuracy (Eq. 1), labeling propensity (Eq. 2), and pairwise correlations of labeling functions (Eq. 3). Accuracy is defined by the correctness of the generated label by the labeling function. In Eq. (1), *p* iterates over tweets and *q* is an iterator of labeling functions. *y* denotes the correct label. So, the equation captures the correct labels each labeling function generates.

1 $$\begin{aligned} \phi _{p,q}^{Acc}(\Lambda , Y)=1\left\{ \Lambda _{p,q} = y_{p} \right\} \end{aligned}$$

Labeling propensity denotes how often a labeling function actually creates a label. In Eq. (2), it is evident that we are considering the instances where a labeling function does not assign an empty label to a tweet.

2 $$\begin{aligned} \phi _{p,q}^{Lab}(\Lambda , Y)=1\left\{ \Lambda _{p,q}\ne \theta \right\} \end{aligned}$$

Pairwise correlations of labeling functions check whether two labeling functions assign the same label to a tweet or not. In Eq. (3), p iterates over tweets where q and r are two different labeling functions.

3 $$\begin{aligned} \phi _{p,q,r}^{Corr}(\Lambda , Y)=1\left\{ \Lambda _{p,q} = \Lambda _{p,r} \right\} (q,r)\in S \end{aligned}$$

All the vectors of these three factors for all *L* labeling functions and correlations *S* are concatenated and represented as $\phi _{p}(\Lambda , Y)$. Now the generative model can be defined as:

4 $$\begin{aligned} \rho _{z}(\Lambda , Y)= U_{-1}^{z}exp \left( \sum _{p=1}^{M}\sum _{t\in T}\sum _{q=1}^{L}w_{q}^{t}\phi _{q}^{t} (\Lambda _{p},y_p)\right) \end{aligned}$$

Here, *T* is the set of dependency types, *U* is the normalizing constant. As we do not know the true labels *Y*, the model learns by estimating the parameter *z* for the distribution of the labeling function output labels Y, given the label matrix, $\Lambda$, the negative log marginal likelihood is minimized. The generative model approximates a posterior distribution of the labeling function outputs while the prior is the ground-truth labels.

5 $$\begin{aligned} {\hat{z}}=arg min_{z} - log\sum _{Y}\rho _z(\Lambda , Y) \end{aligned}$$

This objective function is optimised which produces final predictions, ${\tilde{Y}} = \rho _{{\hat{z}}}(Y \mid \Lambda )$. These we use as final training labels.

#### Discriminative task

**Figure 6:**
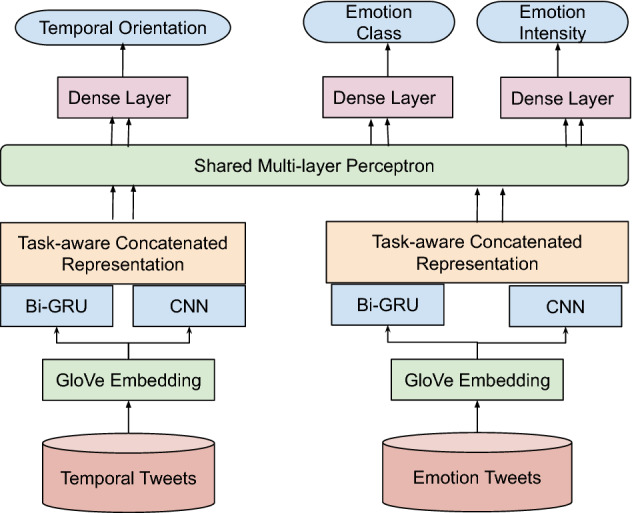
Proposed multi-task discriminator.

The discriminator is a multi-task framework where we simultaneously classify temporal orientation and emotion class. The input to the multi-task discriminator is automatically labeled temporal training tweets along with the existing emotion training tweets. Our multi-task framework uses hard parameter sharing for learning representations. We depict the architecture in Fig. 6.

Both temporal orientation and emotion tweets are vectored using a pre-trained GloVe embedding^70^ of 200 dimensions which were trained on 2 billion tweets. The tweet vectors for both the tasks are then given as input to a Bidirectional-GRU (Bi-GRU)^71^ as well as a Convolution Neural Network (CNN)^72^. The outputs of Bi-GRU and CNN are concatenated, and this is then fed to a shared Multi-Layer Perceptron (MLP) layer. This MLP layer represents the hard parameter sharing of the multi-task framework. The output of the MLP layer is passed through an individual dense layer and then subjected as an input to two different Softmax classifiers for the prediction of two tasks.

#### Model parameters

We perform a grid search to find the optimized parameters for training. The grid search takes all possible combinations of hyper-parameters and selects the best possible combination based on the accuracy of the validation set (10% of the training set). We finalize the parameters as follows: batch size as 64, epochs as 100, loss function as categorical cross-entropy, rms-prop optimizer; CNN filter size as 7, and dropout as 0.2.

### User-level measurement

After finalizing our multi-task model, we use it to predict the temporal orientation as well as the emotion (class and intensity) of a large number of tweets of 5191 users. We group these measures over users using Eq. (6).

6 $$\begin{aligned} orientation_{x}(user)=\frac{|tweets_{x}(user)|}{|tweets_{all}(user)|} \end{aligned}$$

Here, $x \in$ { *past, present*, or *future*} when we calculate the user-level temporal orientation and $x \in$ { *joy, sadness, anger*, or *fear*} when we calculate the user-level emotion.

For the intensity prediction, we divide the intensity in four groups, Very Low Intensity (VLI) Emotion ($score < 0.25$), Low Intensity (LI) Emotion ($score >= 0.25 and <0.5$), Moderate Intensity (MI) Emotion ($score >= 0.5 and <0.75$) and High Intensity (HI) Emotion ($score >= 0.75 and <1$). We finally group these measures over the users using Eq. (7).

7 $$\begin{aligned} orientation_{emoInt}(user)=\frac{|tweets_{EI}(user)|}{|tweets_{all}(user)|} \end{aligned}$$

Here, $EI \in$ is the average intensity score for a particular emotion category of a user.

#### Correlation measure

We find the association between the user-level temporal orientation and the emotion using Pearson’s correlation coefficient *r*.

## Datasets

Our dataset contains three sets of tweets, a separate training set of 7102 tweets for both temporal orientation and emotion, a gold standard test set of 741 tweets for temporal orientation, and 4068 tweets for emotion and $\approx$10 million user-level tweets.

### Training set

We create a training set for the temporal orientation without using any hand-labeled annotation using the label optimization method mentioned in “Methods”. Few examples of tweets with the generated temporal orientation labels are shown in Table 3.

**Table 3:** Few training tweets with generated temporal orientation tags.

**Tweet**	**Temporal orientation**
not even ! it was an onslaught of crap tips!	past
stuck at work as ca n’t get the shutter down ( bored !	present
mcconnell and reid are re opening talks, reports	future

We finally select 7102 tweets as training set (equally distributed between *past*, *present* and *future*). We use two sets of the tweet dataset (EI-OC and EI-reg) from SemEval-2018: Task-1^33^ and a tweet covid-19 dataset^61^ for the emotion training set. The Semantic Evaluation Task (SemEval) is a well-known competition for extracting and analyzing different semantic tasks like sentiment analysis, emotion analysis, etc from texts. The EI-OC data has tweets with four emotion classes (joy, sadness, anger, and fear) and emotion intensity value in an ordinal scale (0–3) where no intensity (0), low(1), moderate(2), and high(3) intensity scores are present. As we are considering intensity as a regression task, we convert the ordinal values in a range of [0–1] using the formula $EI-new = EI-oc \times 0.25 + 0.125$ as followed in^73^. The EI-reg data also have tweets with four emotion classes (joy, sadness, anger, and fear) and the emotional intensity values are between 0 and 1. Both the datasets contain 7,102 tweets manually annotated with *joy*, *sadness*, *anger* and *fear* tags and intensity scores. The tweet covid-19 dataset^61^ contains $\approx$132 million English tweets. We have used this dataset as it has four emotion categories as well as intensity value annotated. We have randomly collected samples (equal samples for each emotion category) from this set to create our training set.

### Test set

We use the existing test sets for evaluation. For the temporal orientation we use the test set created by^21^ which consists of 741 tweets manually annotated with *past*, *present* and *future* orientation. The distribution is as follows: past-375, present-164, and future-202. The emotion test sets are taken from SemEval-2018 Task 1: EI-oc, EI-reg^33^ and tweet covid-19 dataset^61^ each consisting of 4068 tweets of joy, sadness, anger, and fear. The distribution for SemEval-2018 Task 1: EI-oc, EI-reg datasets is as follows: joy-1105, sadness-975, anger-1002, and fear-986. For tweet covid-19 dataset, we use equal number of tweets for each class.

### User-level tweets

We use $\approx$10 million tweets of 5191 Twitter users created by Preoţiuc-Pietro et al.^34^ as the user-level tweets.

## Conclusion

In this article, we have reported a large-scale empirical study that shows that the human temporal orientation can concurrently learn from emotions in a multi-task learning framework. For this purpose, we first built a tweet-level temporal orientation training set that did not require any hand-labeled annotations. We did experiments with single-task and multi-task settings. The evaluation shows that the multi-task model achieves better performance than the single-task model. The user-level association reveals that *future* orientation has associations with *joy* and *anger* while *past* orientation is related to *sadness* and *fear*. We also find that emotional intensity also helps in temporal orientation accuracy. We believe that our study will open up more aspects of digital socio-psychological research where human temporal orientation can be studied on a large scale using various social media data. We also hope that it will provide a more generic approach to measure temporal orientation across cultures, regions, etc.

## Data availability

The datasets generated during the current study are available from the corresponding author on reasonable request.
